# Impact of non-thermal plasma surface modification on porous calcium hydroxyapatite ceramics for bone regeneration

**DOI:** 10.1371/journal.pone.0194303

**Published:** 2018-03-14

**Authors:** Yu Moriguchi, Dae-Sung Lee, Ryota Chijimatsu, Khair Thamina, Kazuto Masuda, Dai Itsuki, Hideki Yoshikawa, Satoshi Hamaguchi, Akira Myoui

**Affiliations:** 1 Department of Orthopaedics, Osaka University Graduate School of Medicine, Suita, Osaka, Japan; 2 Center for Atomic and Molecular Technologies, Graduate School of Engineering, Osaka University, Suita, Osaka, Japan; 3 Department of Neurological Surgery, Weill Cornell Medical College, NY, NY, United States of America; 4 Medical Center for Translational Research, Osaka University Hospital, Suita, Osaka, Japan; Kyoto Daigaku, JAPAN

## Abstract

In the physiochemical sciences, plasma is used to describe an ionized gas. Previous studies have implicated plasma surface treatment in the enhancement of hydrophilicity of implanted musculoskeletal reconstructive materials. Hydroxyapatite (HA) ceramics, widely used in bone tissue regeneration, have made great advancements to skeletal surgery. In the present study, we investigate the impact of low-pressure plasma on the interconnected porous calcium hydroxyapatite (IP-CHA) both *in vitro* and *in vivo*. Our results indicate that dielectric barrier discharge (DBD) plasma, when used with oxygen, can augment the hydrophilicity of non-porous HA surfaces and the osteoconductivity of the IP-CHA disc via increased water penetration of inner porous structures, as demonstrated through microfocus computed tomography (μCT) assay. *In vivo* implantation of plasma-treated IP-CHA displayed superior bone ingrowth than untreated IP-CHA. Though plasma-treated IP-CHA did not alter osteoblast cell proliferation, it accelerated osteogenic differentiation of seeded marrow mesenchymal stem cells. *In vitro* X-ray photoelectron spectroscopy (XPS) revealed that this plasma treatment increases levels of oxygen, rather than nitrogen, on the plasma-treated IP-CHA surface. These findings suggest that plasma treatment, an easy and simple processing, can significantly improve the osteoconductive potential of commonly used artificial bones such as IP-CHA. Further optimization of plasma treatment and longer-term follow-up of *in vivo* application are required toward its clinical application.

## Introduction

Reconstruction of bone defects within clinical settings, through both autograft and allograft treatments, has achieved varying degrees of success but warrants concerns of donor site morbidity [1], potential immune responses, and risk of disease transmission [2]. As an attractive alternative to natural bone grafts, biocompatible and osteoconductive synthetic scaffolds have been widely accepted within orthopedic, craniofacial, and dental disciplines [3–5]. Hydroxyapatite (HA) is a commonly used ceramic in bone regeneration due to its analogous chemical composition to the mineral composites of the target tissue [6] and its affinity to integrate with host tissue [7]. The pervasive use of HA in skeletal surgery has directed much effort towards the advancement of HA-based biomaterials through surface modifications in order to reach higher degrees of biocompatibility and osteocoductivity [8–10].

A group of researchers from Osaka University, including some of the present authors, previously developed a fully open interconnected porous calcium hydroxyapatite ceramics (IP-CHA), now an established bone substitute, commercially available in a clinical setting. When implanted in an *in vivo* rabbit model, the IP-CHA increased in mechanical strength with the formation of new bone, reaching up to three times its initial strength just 9 weeks post-surgery [11]. Due to repetitive mechanical stress loading, early bone ingrowth is necessary for early weight bearing [12, 13]. Treatments facilitating rapid bone ingrowth post implantation can significantly improve the clinical efficacy of currently available bone substitutes.

Plasma, a term used to describe an ionized gas in physicochemical science, can be classified as thermal or non-thermal on the basis of its creation. Thermal plasma is widely used as a surface engineering/coating process through which metals and ceramics can be sprayed onto other material. Metal particles such as titanium or silver as well as hydroxyapatite are imparted on dental or orthopedic implants via a thermal plasma spraying technique to enhance their biocompatibility [14]. Conversely, the potential use of non-thermal plasma, with its lower temperature, has been explored in medical applications for direct treatment of living tissues through sterilization, blood coagulation [15], wound healing, and tissue regeneration [16], or for indirect treatment through implantation of plasma-treated materials into living tissues [17, 18]. Recent studies have established potential advantages of non-thermal plasma treatment in biomaterials used for bone and cartilage regeneration [19, 20], however, no studies have focused on biological effect of non-thermal plasma treatment on porous HA bone substitute.

In the present study, we applied non-thermal plasma treatment to IP-CHA and evaluated the potential of this simple and easy modification to enhance the biocompatibility of preexisting materials. Specifically, we assessed its effect both *in vitro* and *in vivo* using rat calvarial model of bone defect repair on hydrophilicity and osteocoductivity of porous HA bone substitute.

## Materials and methods

### Materials

Samples of HA-coated culture plates (BD BioCoat™ Osteologic™, BD Bioscience, San Jose, CA, USA), non-porous HA pellets (CELLYARD™, Pentax, HOYA Technosurgical Inc., Tokyo, Japan), IP-CHA discs (NEOBONE™, Aimedic MMT, Tokyo, Japan) were processed. IP-CHA discs with 75% porosity in volume, an average pore diameter of 150 μm, and an average interpore-connection diameter of 40 μm were provided by Aimedic MMT Co Ltd. The non-porous HA pellet and IP-CHA disc were 5 mm in diameter and 2 mm in height. All plasma-treated materials were exposed to low-pressure plasma for a duration of 30 minutes, excluding non-porous HA pellets, which were exposed for 5 minutes. Corresponding untreated material served as negative controls in all experiments.

Mouse osteoblastic cell line, MC3T3-E1, was obtained from DS Pharma Biomedical (Osaka, Japan).

### Animals

Twelve 7-week-old (300–400 g) male Sprague-Dawley rat (SD rat) were obtained from CLEA Japan Inc. (Tokyo, Japan). Two rats were used for isolation of bone marrow cells, and ten rats were used for *in vivo* implantation study. These all protocols were approved by the Animal Experimentation Committee of Osaka University.

### Experimental setup of low-pressure, low-frequency plasma treatment

**Fig 1:**
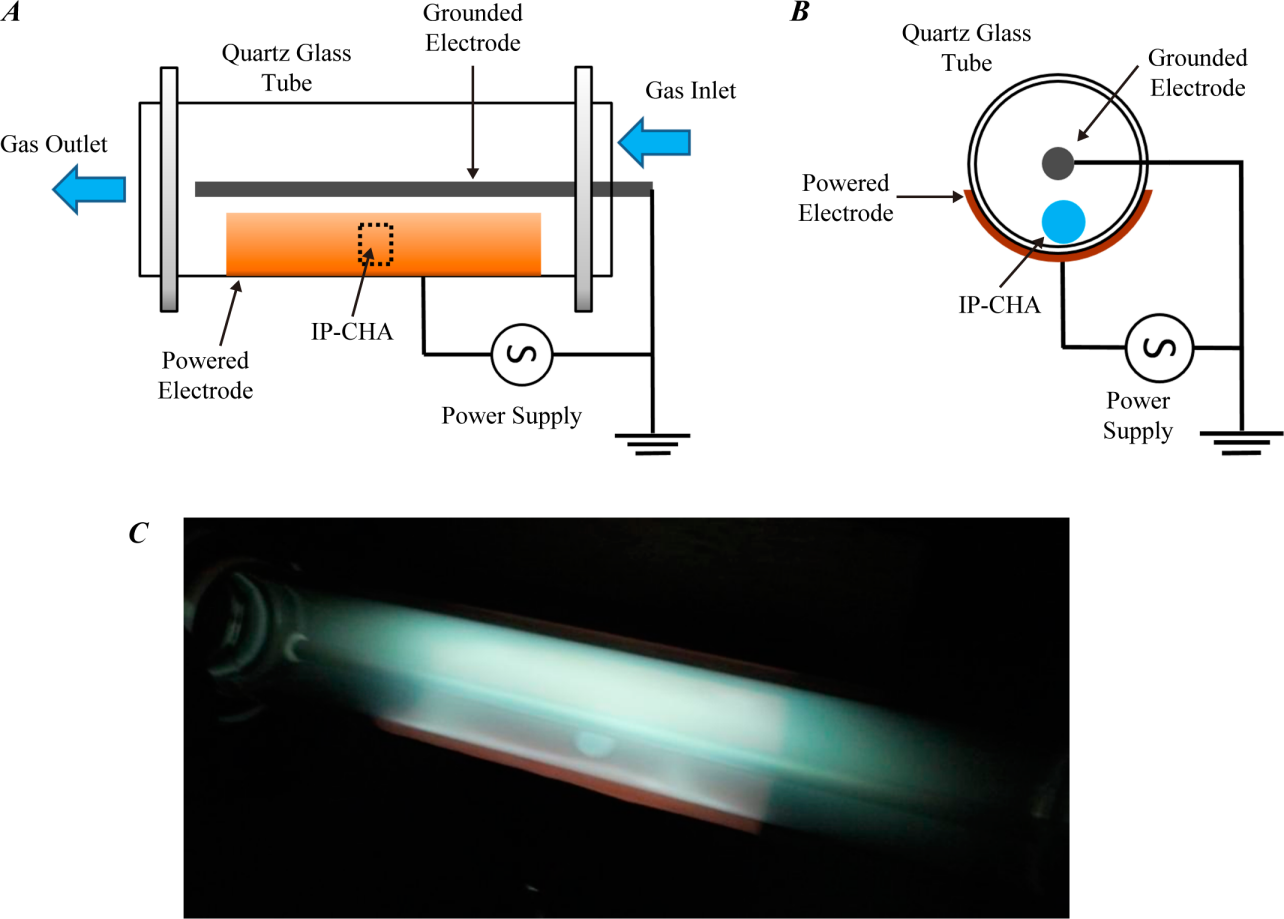
Outline of the experimental settings. Schematic illustration of the discharge system used in this study. A side view (A) of the discharge chamber and its cross section (B). (C) A photograph of a typical discharge with a He/O_2_ gas admixture with a pressure ratio of 5:1, total pressure of 0.6 kPa, an operating voltage (zero-to-peak) of 2.1 kV, and a frequency of 40 kHz. An IP-CHA disc of 5 mm diameter (*φ*) and 2 mm height (*h*) is seen in the discharge chamber.

The plasma treatment was carried out under the same processing conditions as those used in the previous report [21]. Schematic diagrams of the plasma system used presently are shown in Fig 1, where a side view of the discharge chamber along its quartz glass tube, a cross-sectional view of the discharge chamber, and a photograph of a typical discharge are given in (A), (B), and (C), respectively. The discharge system consists of a 12 cm long cylindrical quartz glass tube with inner and outer diameters of 1.7 cm and 2.1 cm, respectively; a powered electrode made of a Cu film placed around the tube, and a metal (SUS) rod inserted at the center of the quartz tube that functions as the grounded electrode. The Cu electrode attached to the outside of the quartz tube was powered by a 40 kHz sinusoidal voltage with an amplitude of 2.1 kV. Individual gases are independently controlled and adjusted to maintain a desired gas pressure whilst the system is pumped by a rotary pump. A photograph (C) illustrates an overall uniform and stable glow discharge formed in a He/O_2_ admixture gas (with the pressure ratio of 5 : 1) at a total pressure of 0.6 kPa.

### Surface wettability of plasma-treated HA

Hydrophilicity of the surface, often considered a measure of biocompatibility of blood-contacting implants, was evaluated using non-porous HA pellets (CELLYARD™, Pentax). The change in hydrophilicity of the HA surface after plasma treatment was characterized by the measurement of contact angles using a drop shape analysis device (PG-X, Matsubo, Japan). Under ambient conditions (50% relative humidity, 21°C), measurements of the contact angles of HA pellets were performed with deionized water as a test liquid immediately after plasma treatment of their surfaces. The surface free energies were subsequently calculated based on the Owens–Wendt–Raabe method.

### Water penetration of porous HA biomaterials treated by low-pressure plasma

To evaluate water penetration of IP-CHA discs, which depends on the hydrophilicity of internal pores, we observed penetration of deionized (DI) water containing a contrast medium (CM, eventually 6% Oypalomin in DI water) into IP-CHA discs using microfocus computerized tomography (μCT). Before the observation by μCT, each IP-CHA disc was entirely immersed in this contrast-enhanced water for 10 minutes and then placed in a μCT system (SMX-100CT-SV; Shimazu, Kyoto, Japan). The x-rays were shielded by a brass film placed in front of the radiation source, eliminating interface scattering and beam hardening effects. Three-dimensional (3D) image data showing the locations of the CM solution in an IP-CHA disc were reconstructed from 2D μCT data with the use of the TRI3DBON software package (Ratoc System Engineering, Tokyo, Japan). For quantitative analysis, we defined the penetration fraction *p* of the CM solution as *p* = 100 × *V*_CM_ / *V*_P_, where *V*_CM_ and *V*_P_ represent the volume of the CM solution and the volume of pores inside the IP-CHA disc [20].

### *In vitro* cell proliferation

Cell proliferation on HA coated surfaces was measured using the WST-8 [2-(2-methoxy-4-nitrophenyl)-3-(4-nitrophenyl)-5-(2,4-disulfophenyl)-2Htetrazolium, monosodium salt] assay with Cell Counting Kit-8 (Dojindo, Kumamoto, Japan). MC3T3-E1 cells were cultured in a non-osteogenic standard medium on untreated and plasma-treated HA-coated culture plates (BD BioCoat™ Osteologic™) at an initial density of 3 × 10^3^ cells per well. The non-osteogenic standard medium consisted of α Eagles’s minimal essential medium (αMEM, Gibco, ThermoFisher Scientific, Waltham, MA, USA) containing 10% fetal bovine serum (Lot No.002095, JRH Bioscience, Lenexa, KS, USA), and antibiotics (100 U/ml of penicillin, 100 μg/ml streptomycin, and 0.25 μg/ml of amphotericin B; Sigma-Aldrich, St. Louis, MO, USA).

At 1, 2, and 4 day time points, 10 μL of the Cell Counting Kit solution was added into each well, followed by incubation of the culture plates at 37°C in 5% CO_2_ for 2 hours. The absorbance was read at 450 nm with an automatic enzyme-linked immunosorbent assay reader (Multiskan Ascent MS353; Thermo Fisher Scientific).

### Combination of rat mesenchymal cells (MMCs) and IP-CHA disc

Rat marrow mesenchymal cells (MMCs) were obtained from the bone shaft of the femora of 7-week-old Sprague-Dawley male rats. After sacrifice with CO_2_ inhalation, both ends of the femur were removed from the epiphysis; the marrow was flushed out using 10 mL of the aforementioned standard medium expelled from a syringe through a 21-gauge needle according to the method developed by Maniatopoulos et al [22]. The released cells were collected in two T-75 flasks (Corning, NY, USA) containing 15 mL of the standard medium. The medium was changed after 24 h to remove hematopoietic cells, and renewed three times a week. Cultures were maintained in a humidified atmosphere of 95% air with 5% CO_2_ at 37°C. After 7 days in primary culture, adherent MMCs were released from the culture substratum using 0.1% trypsin. The cells were concentrated by centrifugation at 900 rpm for 5 min at 4°C and resuspended at 10^6^ cells/mL. The untreated and plasma-treated IP-CHA discs were soaked in 4 mL of cell suspension (10^6^ cells/ml) overnight in a CO_2_ incubator. After the overnight incubation, these MMCs/IP-CHA composites were transferred into a 24-well plate (Corning) for subcultures. DNA content of the MMCs/IP-CHA composite was measured as a cell proliferation assay with IP-CHA discs. Each MMCs/IP-CHA composite was subcultured for 7 days in the non-osteogenic standard medium. After crushing and sonicating each composite, total cellular DNA was extracted using DNeasy Tissue Kit (QIQGEN, Valencia, CA, USA) according to the product manual, and the DNA concentration of the lysate was determined by measurement of the absorbance at 260 nm (A260) in a spectrophotometer (Nanodrop 2000, Thermofisher scientific).

### *In vitro* osteogenic differentiation

To evaluate how the untreated and plasma-treated IP-CHA discs support *in vitro* osteogenic differentiation of MMCs, we subcultured each MMCs/IP-CHA composite in osteogenic medium supplemented with 10 mM of β-glycerophosphate, disodium salt, pentahydrate (Calbiochem, Darmstadt, Germany), 82 μg/ml of _L_-ascorbic acid phosphate magnesium salt *n*-hydrate (Wako Pure Chemical Industrials, Osaka, Japan), and 10^−8^ M of dexamethasone (Dex, Sigma-Aldrich). The medium was renewed three times a week, and the subculture was maintained for 2 weeks. At the end of each subculture period, these MMCs/IP-CHA composites were washed twice with phosphate-buffered saline (PBS), prepared for the staining with alkaline phosphatase (ALP), and evaluated for ALP activity. In the evaluation of the efficacy of *in vitro* osteogenesis, the subcultures in the non-osteogenic standard medium were referred to as ‘no osteogenic induction’. ALP activity was measured as reported previously [23, 24]. In brief, each MMCs/IP-CHA composite was crushed, homogenized in 0.2% Nonidet P-40 containing 1 mM MgCl_2_, and centrifuged at 10,000 rpm for 1 min at 4°C. The supernatant was assayed for ALP activity using *p*-nitrophenyl phosphate as a substrate. ALP was represented as μmol of p-nitrophenol released per composite for 30 min of incubation at 37°C. The total protein content of those samples, which reflects the cell number, was measured by Bio-Rad Protein Assay (Bio-Rad, Hercules, CA, USA) to standardize the ALP activity values.

### Implantation of IP-CHA in rat calvarial defects

Ten 7-week-old (300–400 g) Sprague-Dawley male rats used in this study were handled in accordance with a protocol approved by the institutional ethical committee. All surgeries were performed under sterile conditions in an animal laboratory surgical suite. Under anesthesia with intraperitoneal injection of a mixture of 0.5 mg/kg medetomidine, 4.0 mg/kg midazolam, and 5.0 mg/kg butorphanol, two full-thickness bone defects with a diameter of 5 mm were created symmetrically in the dorsal part of the parietal bone lateral to the sagittal suture using a trephine bur without dura perforation [25]. The plasma-treated and untreated IP-CHA discs were implanted in the right and left holes, respectively [21]. Five rats were sacrificed at 4 weeks, and another five at 8 weeks with CO_2_ inhalation. Histology and μCT analysis were performed to evaluate new bone formation in the inner pores.

### X-ray photoelectron spectroscopy (XPS) analysis

X-ray photoelectron spectroscopy (XPS) analysis was performed using ESCA-850 (Shimadzu, Kyoto, Japan) in order to determine the chemical composition of IP-CHA after plasma treatment [26, 27]. The X-ray source of the XPS system was a magnesium anode biased at 8 kV with a current of 30 mA, which emits non-chromatic Mg K_α_ line with a photon energy of 1,253.6 eV and an energy spread of 0.7 eV. The pass energy for photoelectrons was set at 75 eV with an photoelectron energy analyzer resolution of 0.1 eV. The pressure in the XPS analysis chamber was set below 2.0 × 10^−6^ Pa. Prior to each XPS measurement, surface contamination (mostly by carbon) was removed by Ar-beam etching for 2 seconds in the XPS system. Since the diameter of collimated X-ray irradiation region is about 8 mm, the nearly entire area of the IP-CHA surface facing the X-ray source is exposed to the X-ray. Therefore the XPS system provides data averaged over the sample surface. The relative surface concentration of each chemical element was determined from the corresponding high-resolution narrow-scan spectrum, using symmetrical Gaussian peak shapes and integrated background subtraction. The peak positions were determined in the curve fitting process according to tabulated chemical shifts [28]. XPS measurements were performed for multiple IP-CHA samples of the same nature in each category to ensure statistical significance of the obtained data.

### Microfocus computed tomography (μCT)

To evaluate the penetration of CM *in vitro* and the formation of new bone *in vivo* in each IP-CHA disc, we used μCT system (SMX-100CT-SV; Shimadzu). Each sample was scanned at 10 μm intervals at 50 kV and 200 μA. Analysis was performed in superprecision mode at seven times magnification, with an image intensifier of 1.8 inch in diameter. After scanning, 3D-CT images were reconstructed, and a discoid image (φ5 mm, 2 mm height) was extracted from the center of each IP-CHA disc. An additional discoid image (φ2.5 mm, 0.67 mm height) of each *in vivo* sample was extracted from the innermost twelfth region (with a half diameter and a one-third height of the implant ) and the bone formation close to the core of the implant was evaluated. The volume of CM- or bone-filled pores was measured using the TRI3DBON Software package (Ratoc System Engineering, Tokyo, Japan).

### Histological evaluation

Decalcified sections were obtained in the following manner; 5 implants harvested from each group at 4 and 8 weeks were fixed in 10% buffered formalin, decalcified in K-CX solution (Falma Co., Tokyo, Japan), dehydrated through ethanol series, and embedded in paraffin. 4 μm thick sections were cut parallel to the anatomical coronal planes through the axes of IP-CHA discs, then stained with hematoxylin and eosin (HE) for light microscopy.

### Statistical analysis

Statistical analysis was performed with unpaired t-test with software, STATVIEW version 4.5 (SAS Institure Inc., Cary, NC). The statistical significance level was set at *p* = 0.05.

## Results

### Surface wettability of non-porous HA treated by low-pressure plama

**Fig 2:**
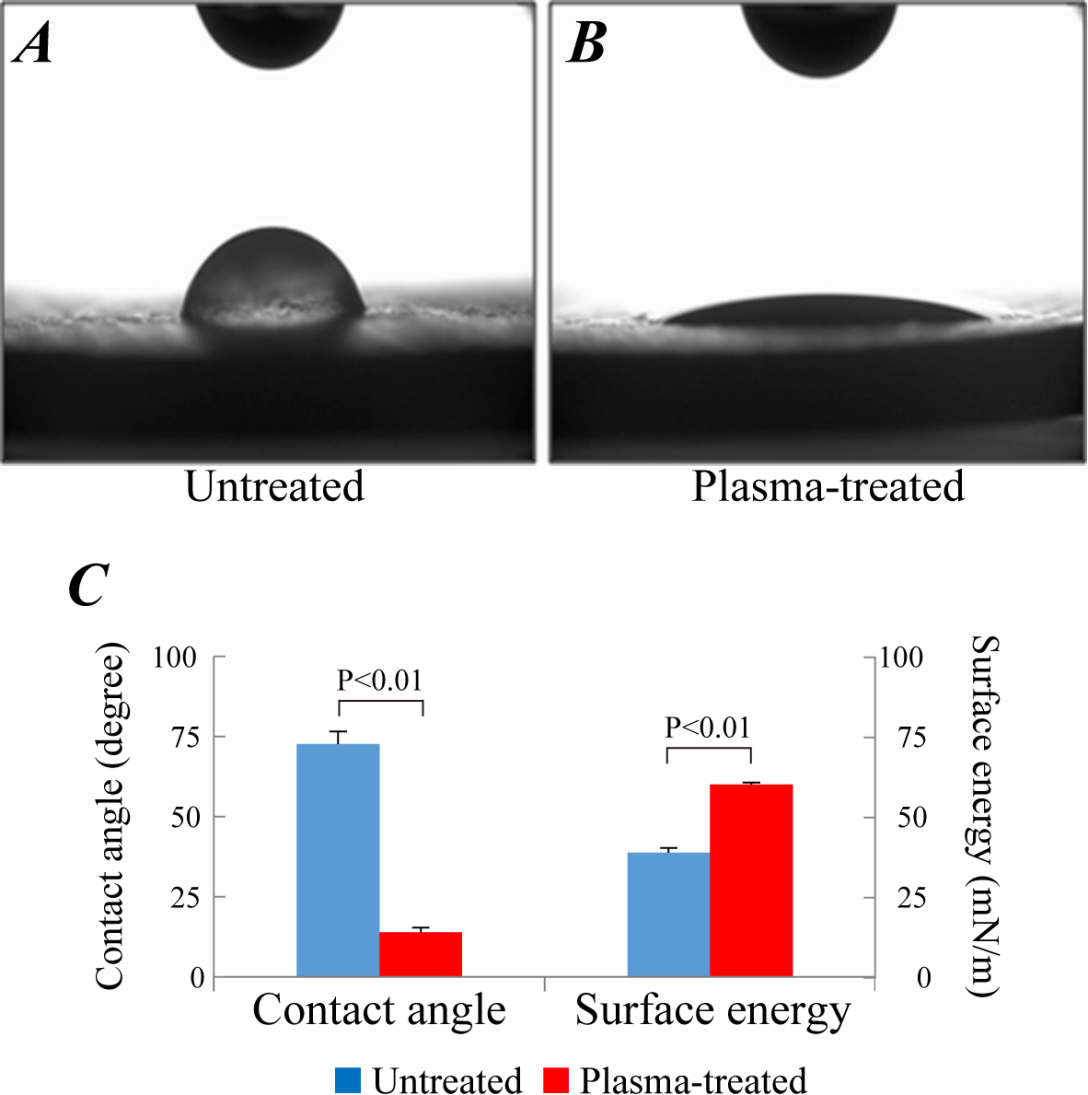
Change of surface wettability after 5 min of plasma treatment. Photographs of water drops on the surfaces of non-porous HA pellets. (A) untreated and (B) plasma-treated pellets. (C) Quantitative analysis of contact angles and surface energy. Data were expressed as mean ± standard deviation (SD).

Water drops placed on non-porous HA surfaces treated with plasma for a duration of 30 minutes spread immediately and their contact angles were too small to be measured. The plasma exposure was therefore reduced to 5 minutes and optimized for contact angle assessment. Photographs of water drops are displayed in Fig 2A and 2B; quantitative analysis in Fig 2C indicates significant difference in the contact angle and surface free energy between the untreated and plasma-treated group (72.6 ± 3.89° vs 13.8 ± 1.43°, p < 0.05) (38.8 ± 1.42 mN/m vs 60.1 ± 0.51 mN/m, p < 0.05). The decrease of contact angle and increase of surface energy verifies that a low-pressure plasma treatment leads to a significant increase in hydrophilicity of the non-porous HA.

### Water absorbability of inner porous HA biomaterials using μCT analysis

**Fig 3:**
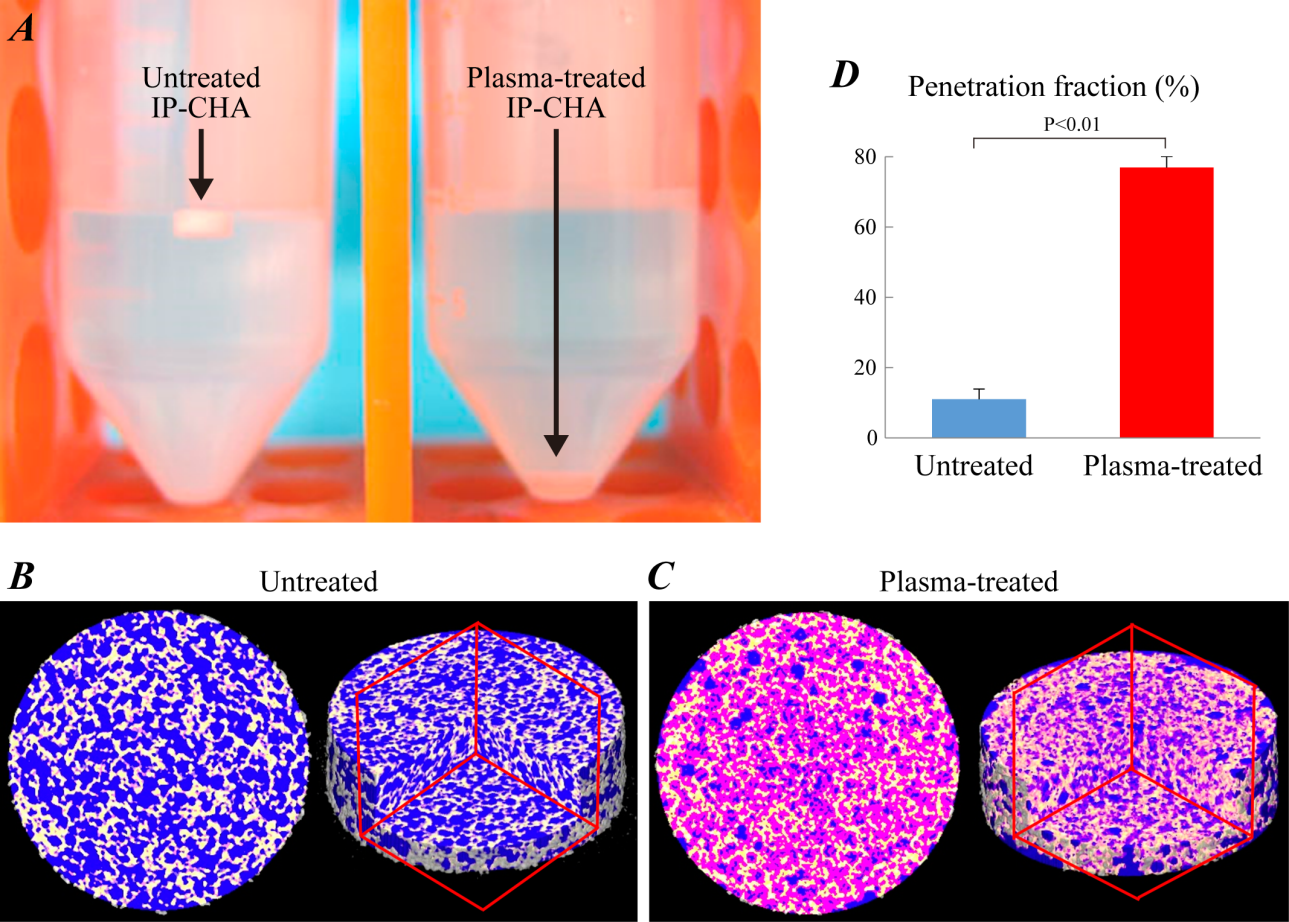
Analysis of water penetration into IP-CHA. (A) Photographs of untreated and plasma-treated IP-CHA discs (*φ*5 mm × *h*2 mm) soaked in the deionized water containing 6% contrast medium (CM, Oypalomin™). Both discs were soaked in the CM solution for 10 minutes, and evaluated by μCT regarding penetration of CM solution into inner pores. The μCT cross section at half height (left) and 3D (right) images of untreated (B) and plasma-treated IP-CHA discs (C). Each image is composed of three parts: yellow for HA, blue for empty space (i.e. unfilled pores) and pink for the CM solution (i.e. CM filled pores). (D) Penetration fraction of inner pores was calculated. Data were expressed as mean ± SD.

The untreated discs remained afloat in water after 10-minute soaking (Fig 3A). By contrast, plasma-treated discs submerged in water immediately, indicating increased water absorbability, which is dependent on hydrophilicity of inner pore surfaces. Both discs were subsequently evaluated by μCT regarding the penetration of a CM solution into the inner pores. The μCT 3D images of untreated (Fig 3B) and plasma-treated IP-CHA discs (Fig 3C) are shown in Fig 3. Each image constitutes three parts; yellow for HA, blue for empty space (i.e. unfilled pores) and pink for the CM solution (i.e. CM filled pores). With plasma treatment, most pores became filled with the CM solution, supporting the increase in hydrophilicity of inner pore surfaces. Quantitative analysis further demonstrated that the penetration fraction of inner pores was significantly increased by the plasma treatment (Fig 3D, 10.9 ± 2.97% vs 76.9 ± 3.19%, p < 0.05).

### *In vitro* cell proliferation analysis

**Fig 4:**
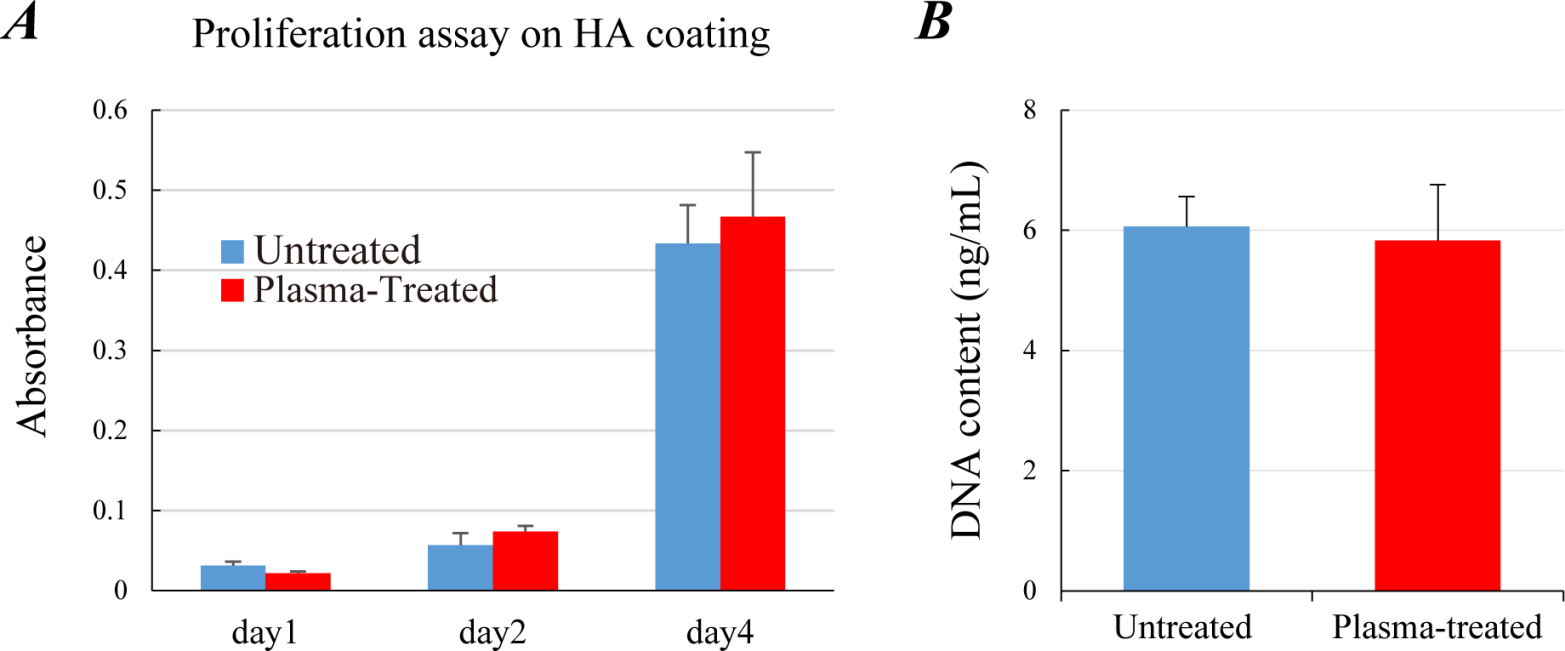
Cell proliferation on HA surfaces in non-osteogenic culture. (A) WST-8 assay for MC3T3 cultured on HA-coated plates at day 1, 2 and 4. Blue and red lines denote untreated and plasma-treated groups, respectively. (B) DNA content of IP-CHA discs (*φ*5 mm × *h*2 mm) combined with rat MMCs. The MMCs/IP-CHA composites were cultured in non-osteogenic growth medium for 7 days. Data were represented as mean ± SD of 2 independent triplicate experiments.

The WST-8 assay using MC3T3-E1 cells and HA-coating plates demonstrated no significant difference in cell proliferation between the untreated and plasma-treated groups at each time point (Fig 4A). IP-CHA discs combined with rat marrow mesenchymal cells (MMCs) were cultured in non-osteogenic growth medium for 7 days, and DNA content of these MMCs/IP-CHA composites were measured. Likewise, no significant difference was found in the DNA content per composite between two groups (Fig 4B). Data in Fig 4 were represented as mean values ± standard deviation of 2 independent triplicate experiments.

### *In vitro* osteogenic differentiation of MMCs / IP-CHA composites

**Fig 5:**
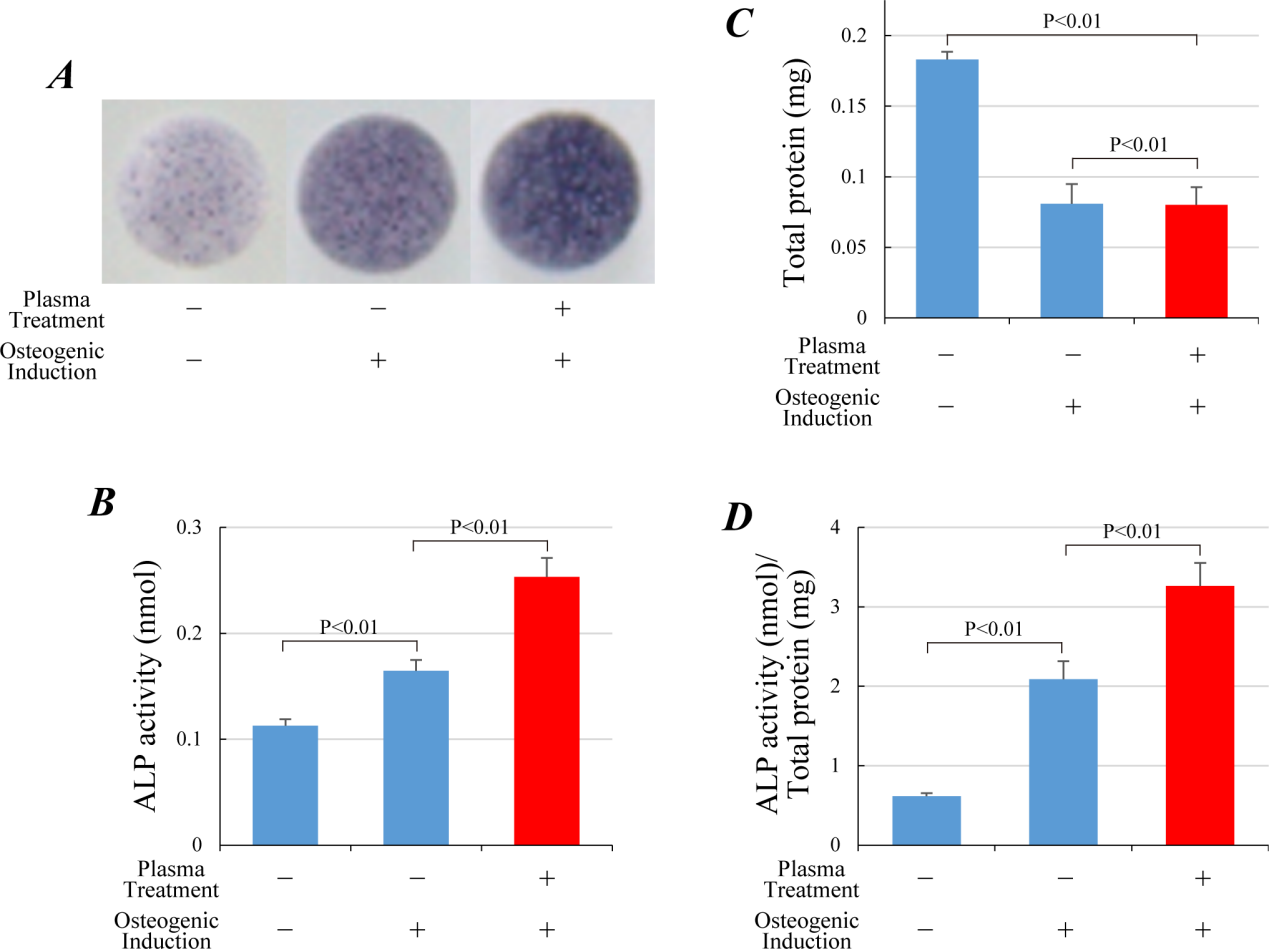
*In vitro* osteogenesis of IP-CHA discs combined with rat MMCs. (A) Alkaline phosphatase (ALP) staining of IP-CHA discs, which were combined with MMCs and cultured in vitro for 14 days in non-osteogenic or osteogenic differentiation medium. Each graph is composed of three groups: no osteogenic induction (left), plasma-untreated (center), and plasma-treated (right) IP-CHA discs. ALP activity (B) and content of total protein (C) per MMCs/IP-CHA composite. (D) ALP activity normalized by the content of total protein. Data were represented as mean ± SD of 2 independent triplicate experiments.

After the osteogenic subculture for 14 days, the MMCs/IP-CHA composites were evaluated. ALP staining was more intensively detected in the osteogenic-differentiated group (as seen in ‘untreated’ and ‘plasma-treated’ of Fig 5A), with further enhancement in the plasma-treated group. Quantitative analysis demonstrated that ALP activity was highest in the plasma-treated group (Fig 5B: 0.165 ± 0.010 in ‘untreated’, and 0.253 ± 0.018 in ‘plasma-treated’). Conversely, the total protein content of the composites was significantly lower under osteogenic differentiation without any difference between ‘untreated’ and ‘plasma-treated’ groups (Fig 5C: 0.165 ± 0.010 in ‘untreated’, and 0.253 ± 0.018 in ‘plasma-treated’). This suggests that, unlike the ‘no osteogenic induction’ group subcultured in the standard medium, the MMCs in the other two groups despite not proliferating intensely, differentiated into osteogenic lineages. Accordingly, the standardized ALP activity divided by the corresponding total protein content significantly increased in the plasma-treated group as compared with the untreated group and the group of ‘no osteogenic induction’. (Fig 5D: 0.617 ± 0.038 in ‘no osteogenic induction’, 2.089 ± 0.228 in ‘untreated’, and 3.263 ± 0.288 in ‘plasma-treated’).

### X-ray photoelectron spectroscopy (XPS) analysis

To examine the change in surface chemical composition of IP-CHA by plasma treatment, we have performed XPS measurements of untreated and plasma-treated IP-CHA sample surfaces. The results are summarized in Table 1, where the corresponding theoretical values of stoichiometric HA, i.e., Ca_10_(PO_4_)_6_(OH)_2_, are also listed as a reference. Quadruplicated (n = 4) XPS measurements were performed for untreated IP-CHA samples and triplicated (n = 3) measurements were performed for plasma treated IP-CHA. It is evident that the surface of an untreated IP-CHA used in this study was not stoichiometric HA. The IP-CHA surface had a higher Ca to P atomic concentration ratio (Ca/P) and a lower O to Ca atomic concentration ratio (O/Ca) than the stoichiometric HA, which suggests that IP-CHA consisted of a mixture of stoichiometric HA and other calcium phosphates [26, 27]. It is seen that, by plasma treatment, the mean values of O/P and O/Ca increased from 4.27 and 3.39 to 6.07 and 4.78, respectively, with the corresponding P values of 8.7× 10^−4^ and 8.3 × 10^−3^ (i.e., P < 0.01), indicating that the increase of O atoms on the IP-CHA surface by plasma treatment was statistically significant.

**Table 1:** The surface atomic concentration ratios obtained from XPS.

	Atomic concentration ratios		
	Ca/P	O/P	O/Ca
Stoichiometric HA theoretical values	1.67	4.33	2.60
Untreated IP-CHA	1.21±0.15	4.27±0.26	3.39±0.48
Plasma-treated IP-CHA	1.27±0.05	6.07±0.31*	4.78±0.05*

Atomic concentration ratios of calcium to phosphor (Ca/P), oxygen to phosphor (O/P), and oxygen to calcium (O/Ca) on the surfaces of untreated IP-CHA and plasma treated IP-CHA obtained from XPA measurements. Measurements are quadruplicated (n = 4) for untreated IP-CHA and triplicated (n = 3) for plasma-treated IP-CHA. Data are expressed as mean ± SD. As a reference, the corresponding theoretical values of stoichiometric HA, i.e., Ca_10_(PO_4_)_6_(OH)_2_, are also listed in the first row. It is seen that, in the plasma-treated IP-CHA (n = 3), the relative surface concentration of O increased significantly, whereas the Ca/P ratio remains unaltered.

*P < 0.01; compared to untreated IP-CHA (n = 4).

**Fig 6:**
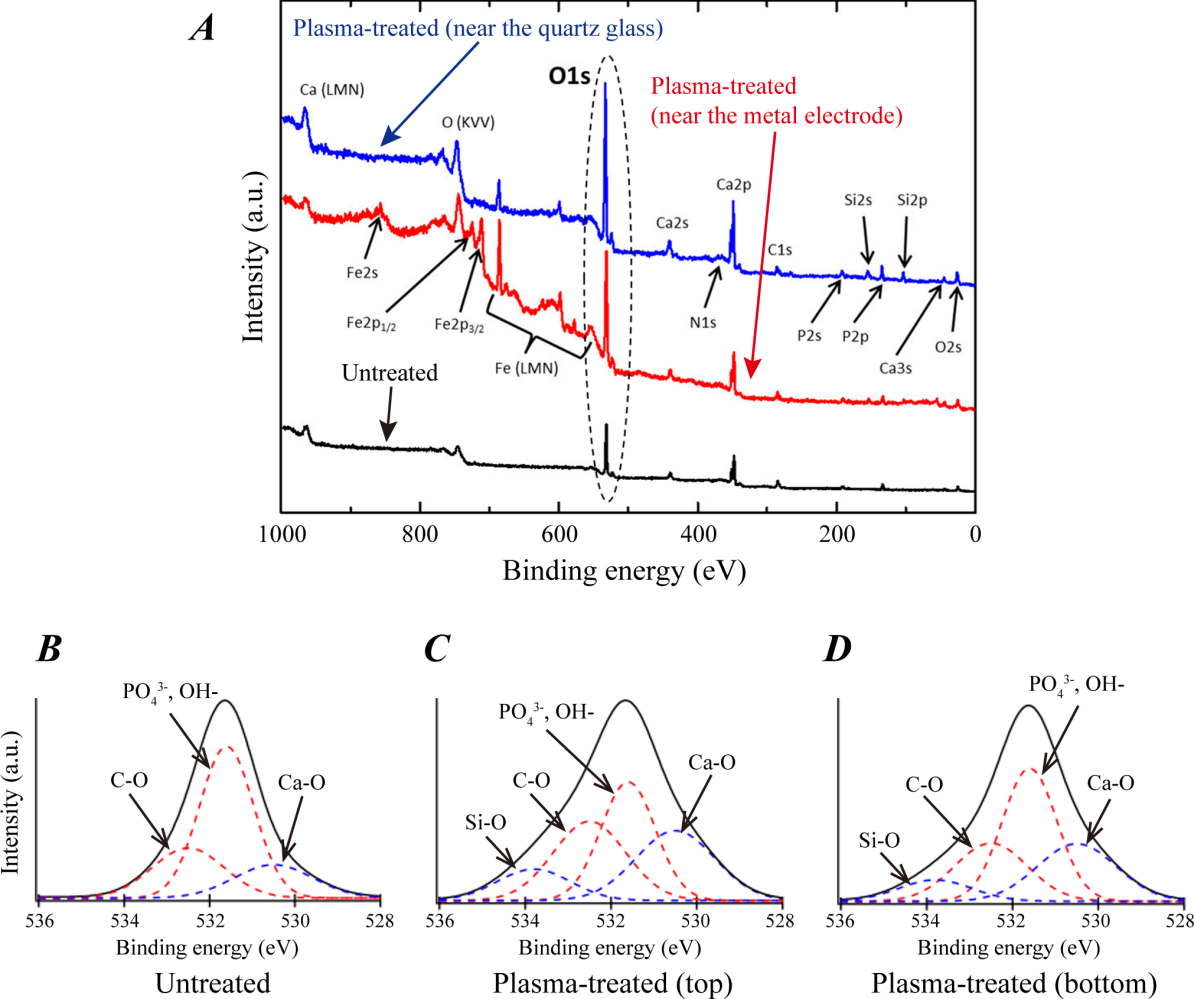
Wide scan XPS analysis of IP-CHA discs. (A) Wide scan spectra. It is seen that the O1s peak of the IP-CHA surface increased notably after plasma treatment (broken oval). The Fe peaks appear on the IP-CHA surface that faced the metal electrode (red), whereas the Si peaks appear after plasma treatment, more significantly so on the surface that faced the quartz glass (blue). (B) ,(C), and (D) are narrow scan spectra of O1s for the IP-CHA surface without plasma treatment, the top surface of an IP-CHA disc with plasma treatment, and the bottom surface of an IP-CHA disc with plasma treatment, respectively. We consider that C is a contaminant and Si comes from the quartz glass wall of the discharge chamber.

Knowing that the oxygen content on the IP-CHA surface increased significantly by plasma treatment, we now check if there was any other significant change in surface chemical composition by plasma treatment. Fig 6A shows representative samples of XPS wide scans for a plasma-treated surface of an IP-CHA disc that faced the quartz glass surface during the plasma treatment (the top curve in blue), a plasma-treated surface of an IP-CHA disc that faced the stainless-steel metal electrode during the plasma treatment (the middle curve in red), and an untreated surface of an IP-CHA disc (the bottom curve in black). As indicated by the broken oval in Fig 6A, the O1s peak of the IP-CHA surface increased notably after plasma treatment, in agreement with our discussion above. It is also seen that the Fe peaks appeared on the IP-CHA surface that faced the metal electrode, whereas the Si peaks appeared on the surface that faced the quartz glass. It is most likely that Fe atoms on the IP-CHA surface were originated from the stainless steel electrode sputtered by the plasma, whereas Si atoms on the IP-CHA surface were originated from the quartz wall also sputtered by the plasma. Although signals of Fe and Si are seen in XPS, these peaks are known to be relatively sensitive (i.e., large even when a small amount of the corresponding species exists) and we have confirmed that the atomic percentages of Fe and Si were negligibly small. Similarly a small amount of carbon is observed in both untreated and plasma-treated IP-CHA and hardly affects the atomic concentration ratios among Ca, P, and O, i.e., constituting atoms of HA, which suggests the observed carbon is an inconsequential part (most likely contamination by ambient air) of the IP-CHA surface. We also note that no nitrogen peak is seen in either untreated or a plasma-treated surface. Thus we have concluded that the only confirmed significant change in surface chemical composition of IP-CHA by plasma treatment is the increase of O atoms.

Fig 6B–6D also shows sample narrow scan spectra of O1s for the IP-CHA surface in untreated IP-CHA (Fig 6B), the top surface of a plasma-treated IP-CHA disc (Fig 6C), and the bottom surface of a plasma-treated IP-CHA disc (Fig 6D), corresponding to the three cases shown in (Fig 6A). The spectra were fitted with 4 possible chemical shifts, i.e., C-O, Si-O, Ca-O, and phosphate or hydroxyl ions (i.e., PO_4_^3-^ or OH^-^). The Ca-O chemical shift seems to have increased after plasma treatment, but it is not conclusive from these findings here. A further study is needed to determine how the increased oxygen is bonded in the plasma treated IP-CHA.

### μCT analysis of newly formed bone volume

**Fig 7:**
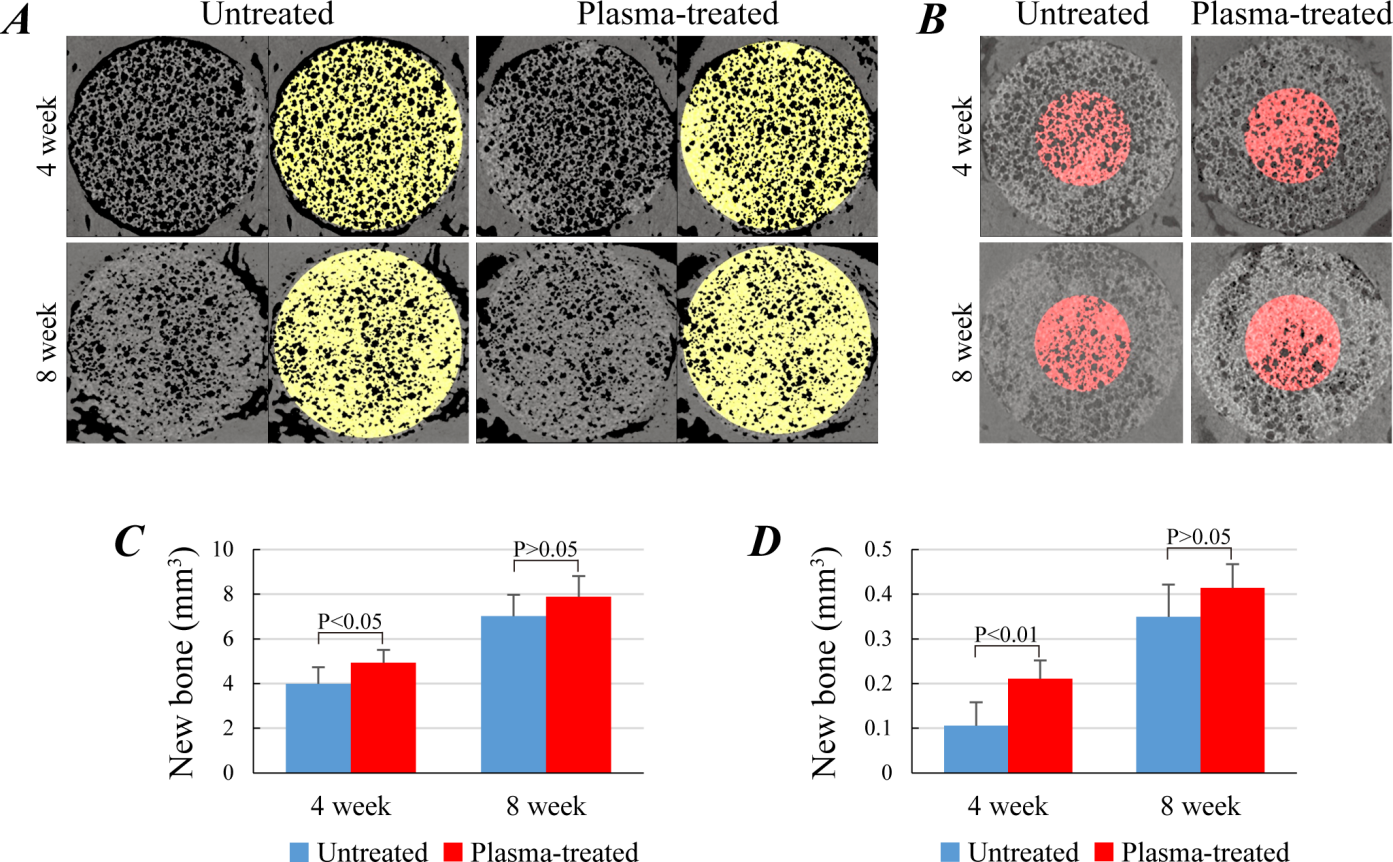
*In vivo* implantation of IP-CHA discs. (A) Images of μCT analysis of untreated and plasma-treated IP-CHA discs at 4 and 8-week time points after implantation. Yellow denotes the bony density (i.e. IP-CHA and newly-formed bone inside the pores). (B) Pink denotes the bony density in the discoid images (φ2.5 mm, 0.67 mm height) extracted from the innermost twelfth part of the implant. Quantitative analysis of newly formed bone in the whole (C) and the innermost part (D) of the IP-CHA disc. Data were represented as mean ± SD of 5 animals.

To measure the total bone volume in the plasma-treated/untreated IP-CHA discs after *in vivo* implantation, we performed μCT analysis as previously reported [24]. In Fig 7, the μCT images display areas having bony high (yellow) and low (black) intensities (Fig 7A). The yellow region, representing the wall of IP-CHA and bone matrices newly formed in the inner pores, was larger in the plasma-treated group than in untreated at 4 weeks, with a slight difference at 8 weeks. This potential superiority of plasma-treated group in osteoconductivity is mo.

**Fig 8:**
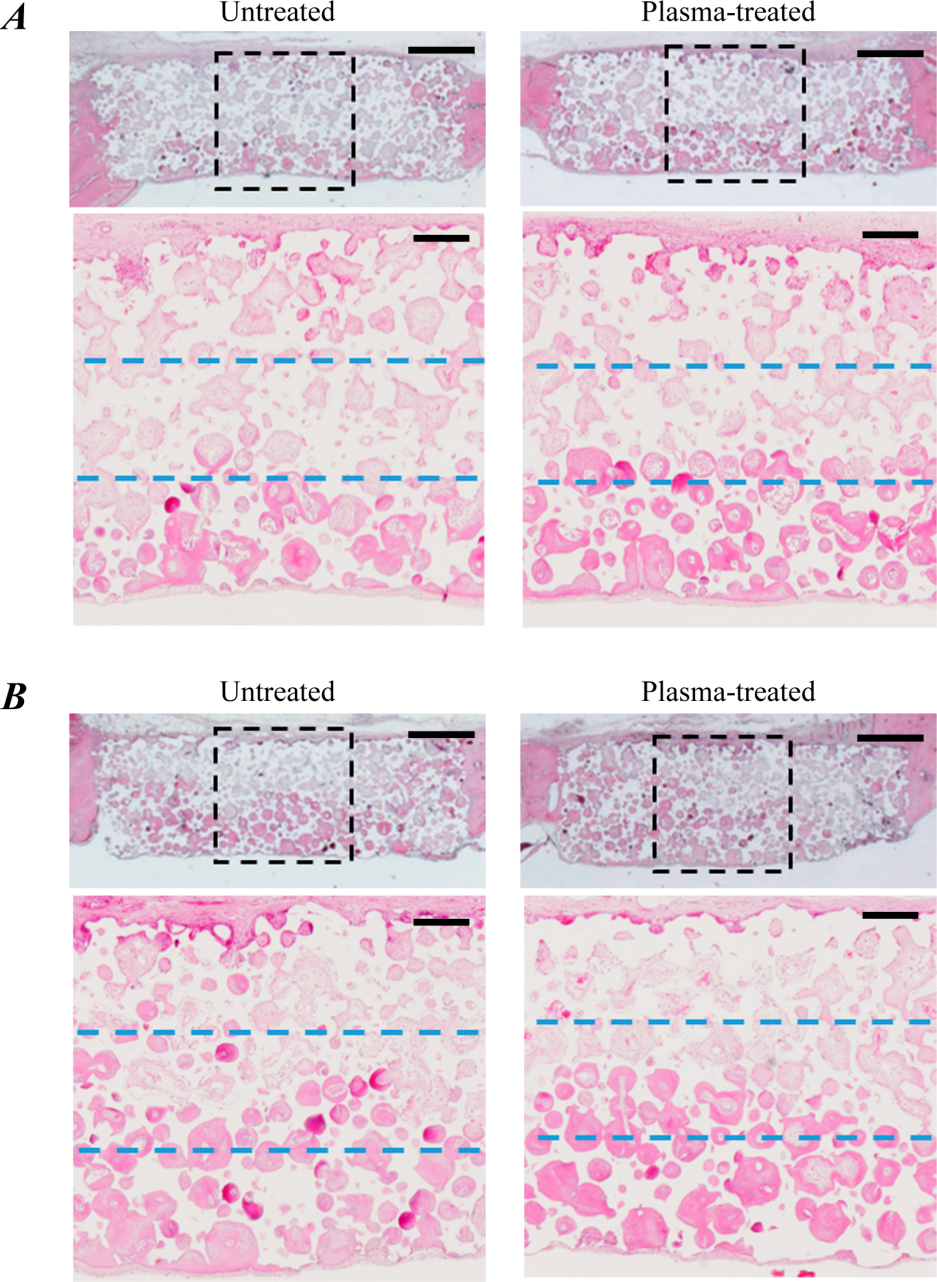
Histological images of IP-CHA discs after *in vivo* implantation. Hematoxylin & Eosin-staining of IP-CHA discs at 4 and 8 weeks after *in vivo* implantation (A and B respectively). Scale bars = 200 μm. At both time points, the bone formation in the pores of the implants was observed in the bottom region close to the brain surface, which is known to be rich in blood supply; less bone was produced with increasing distance from the bottom region to the top surface of the implant. Overall, the plasma-treated discs had more bone formation in the pores compared to untreated controls (top in A&B). In the close-up images (bottom in A&B), the plasma-treated discs demonstrated bone ingrowth even in the core region at 4 weeks, while the untreated controls did not. At 8 weeks, the progress of bone ingrowth reached the core regions of both untreated and plasma-treated implants, with the plasma-treated implants having slightly more bony tissue.

Quantitative analysis demonstrated that plasma-treated IP-CHA had significantly higher bone volume than untreated IP-CHA at 4 weeks (Fig 7C: 3.98 ± 0.75 mm^3^ vs 4.93 ± 0.57 mm^3^, P < 0.05). However, the bone volume at 8 weeks did not reach statistical significance (Fig 7C: 7.01 ± 0.97 mm^3^ vs 7.88 ± 0.93 mm^3^, P > 0.05). Additionally, between the two groups, the volume of new bone in the innermost twelfth region had more distinctive difference, which reached the statistical significance at 4 weeks but not at 8 weeks (Fig 7D). These data were consistent with the results of histological analyses shown in Fig 8.

### Histological analysis after *in vivo* implantation

At 4 and 8 weeks (Fig 8A and 8B, respectively), the bone formation in the pores of the implants was observed in the bottom region close to the brain surface, which is known to be rich in blood supply; less bone was produced with increasing distance from the bottom region to the top surface of the implant. At both time points, the plasma-treated discs had more bone formation in the pores compared to the untreated controls (top in Fig 8A and 8B). To closely observe the ingrowth of bony tissue, we magnified the center of each disc (approximately one-third of the disc diameter) and drew blue dashed lines at one-third and two-thirds of the disc’s height (bottom in Fig 8A and 8B). Therefore, the region between the blue dashed lines is the core of the interconnected porous implant and farthest from its outer surface. In the plasma-treated group, the bone formation inside the pores of IP-CHA was visible even in the core region at 4 weeks. However, essentially no bone formation was observed in the core region of the untreated group at 4 weeks. Further, the bone ingrowth progression was steadily upward and reached the core region at 8 weeks in both untreated and plasma-treated implants, with the plasma-treated implants having slightly more bony tissue.

## Discussion

Among various surface modification approaches available for biomaterials, plasma treatment has many attractive features; it is solvent-free, has low chemical consumption, and requires no sterilization [29]. A variety of plasmas with varying excitation methods from different gas sources have been used to improve physical and biological properties of target material surfaces [18, 30, 31]. In fact, plasma treatments for HA surfaces have been well reported in literatures, but these studies are limited to atmospheric plasma or low pressure ion implantation using radio frequency plasma [19, 32]. The present study is the first to demonstrate the feasibility of low-pressure and low-frequency plasma to process a HA-based porous material and facilitate its osteoconductivity.

Osteoconduction of an implant surface, i.e., the process that guides the growth of a bony tissue directly bound to the surface, is closely associated with the susceptibility of bone ingrowth. It is one of the most important factors in the development of new artificial bone substitutes. Few studies have employed similar strategies to enhance the osteoconductivity of porous HA using different processing techniques, which efficiently facilitate new bone formation on HA surfaces [10, 33]. Recent studies have now revealed that surface modification of HA with plasma, an easy and economical technique, can further improve its surface hydrophilicity and mineralization [19] and penetrate its porous structures via interpore connections [34, 35].

In a previous study, some of the present authors showed that both gas pressure and treatment time affect the efficacy of plasma treatment by facilitating water penetration into a plasma-treated porous material (IP-CHA), and determined the optimal process parameters for low-pressure, low-frequency plasma processes of IP-CHA [21]. Using those parameters in the present study, we discovered that the penetration of contrast-enhanced water into plasma-treated IP-CHA discs (5 mm diameter, 2 mm height) was promoted owing to increased hydrophilicity on the outer surfaces of IP-CHA discs and inner walls of their pores. Immersion of IP-CHA discs in solution, however, can be influenced by trapped air within the pores, which can impede or even prevent further penetration of the solution into the pores unless ventilated or absorbed by the solution. Therefore, the penetration fraction *p* shown in the present study may not completely reflect hydrophilicity of the inner pore surfaces. However, it remains a sound indicator of water absorbability of the porous materials [21].

Plasma treatment can enhance the *in vivo* osteoconductivity of HA-based materials. Although pro-osteogenic modification of HA surfaces was achieved by plasma treatment *in vitro*, no previous study demonstrated its efficacy *in vivo* [19]. In the present study, bone ingrowth into a porous HA was accessed in a rat calvarial defect model. Histological analysis in coronal sections demonstrated better bone ingrowth into IP-CHA discs at the regions close to the epidural space, thus probably rich in blood supply. Quantitative μCT analysis revealed that the new bone volume in the IP-CHA discs significantly increased by plasma treatment at 4 weeks, with no difference between groups at 8 weeks. These results suggest that plasma treatment of IP-CHA facilitates bone ingrowth especially at an early stage of bone healing. Clinically, the effect induced by plasma treatment can rapidly increase the mechanical strength of IP-CHA implants, resulting in early weight bearing and faster rehabilitation.

Interestingly, in the core region which comprises the innermost area with a twelfth volume of the whole, the new bone formation was significantly greater at 4 weeks in the plasma group. This trend of superiority was also observed at 8 weeks, despite not reaching statistical significance. The enhancement in osteoconduction by plasma treatment was most noticeable in the core region than in the whole IP-CHA disc. This is due to the interconnected pores of the core which enables sufficient plasma-induced osteoconductivity. Further, considering that the improvement in the innermost region requires the penetration of fluid and cells, the increased water absorbability of IP-CHA discs by plasma treatment may have strongly affected the *in vivo* experiment. Therefore, the enhanced *in vivo* osteoconduction observed in this study may be due, in part, to the increased water absorbability, along with some pro-osteogenic changes of the material surface [19].

Changes in the surface chemical composition, which may be responsible for the biological response of cells contacting the material, were elucidated by XPS analysis. The observed increase in concentration of oxygen (O) at the surface of IP-CHA is similar to reports in other studies examining plasma treatment of polymer surfaces [19, 36, 37]. Although it is unclear from the XPS data how O atoms were incorporated in the surface atomic structures, we surmise that most O atoms were incorporated as negatively charged hydroxyl (OH) groups near positively charged Ca atoms on the surface, based on the fact that IP-CHA becomes highly hydrophilic after plasma treatment and hydroxyapatite is known to adsorb further hydroxyl ions (OH^-^) in a weakly alkaline electrolyte solution [38]. However a further study is needed to determine surface chemical structures of IP-CHA after plasma treatment. Unlike the previous study using atmospheric plasma [19], no substantial change in the concentration of nitrogen (N) was observed before and after the plasma treatment in this study. This is reasonable as we had no intentional admixture of air in the He/O_2_ plasma. Hence, the difference in findings between our study and the previously published study that implicated nitrogen in the enhanced proliferation of the cells cultured on HA coatings suggests that the efficacy of plasma treatment is highly dependent on the gases used for plasma generation.

Our findings suggested that plasma treatment can enhance the *in vivo* osteoconductivity of IP-CHA through increased water absorbability and pro-osteogenic surface modification. The application of plasma to commonly used artificial bones is a simple and easy modification of preexisting biomaterials that can substantially enhance their bone healing and regenerative properties.

## Conclusions

The present study demonstrated that plasma application to IP-CHA, a widely used bone substitute, enhanced its *in vitro* and *in vivo* performance as a bone substitute. Further optimization of plasma treatment and longer term follow-up of its *in vivo* application will facilitate its use in a clinical setting.
